# Establishing Pain Thresholds for Functional Recovery After Lung Cancer Surgery: A Mediation Analysis of the Surgery–Pain–Functioning Causal Pathway

**DOI:** 10.1155/prm/3783225

**Published:** 2025-11-28

**Authors:** Pan Ma, Wei Dai, Shizhu Li, Rumei Xiang, Hongfan Yu, Xing Wei, Jia Liao, Cheng Lei, Wei Xu, Xiangxi Zhou, Zhibiao Wang, Qiuling Shi

**Affiliations:** ^1^State Key Laboratory of Ultrasound in Medicine and Engineering, College of Biomedical Engineering, Chongqing Medical University, Chongqing, China; ^2^Department of Pharmacy, First Affiliated Hospital of Army Medical University, Chongqing, China; ^3^Department of Thoracic Surgery, Sichuan Clinical Research Center for Cancer, Sichuan Cancer Hospital & Institute, Sichuan Cancer Center, Affiliated Cancer Hospital of University of Electronic Science and Technology of China, Chengdu, Sichuan, China; ^4^Department of Public Health, Zigong Forth People's Hospital, Zigong, Sichuan, China; ^5^School of Public Health, Chongqing Medical University, Chongqing, China

**Keywords:** lung cancer surgery, mediation analysis, physical functioning, pain management, pain thresholds

## Abstract

**Objective:**

Pain is one of the most common and long-lasting symptoms after lung cancer surgery, potentially impairing physical functioning. This study aims to investigate to what extent pain mediates the association between the different surgical approaches (single-port VATS versus multiport VATS or thoracotomy) and postoperative functional recovery after lung cancer surgery, with the goal of establishing clinically actionable pain score thresholds.

**Methods:**

In a prospective cohort study including 1381 patients, pain and functional status (activity limitation and walking difficulty) were assessed daily in hospital with the Perioperative Symptom Assessment for Lung Surgery (PSA-Lung). The structural equation model (SEM) was used to investigate the mediation effect of pain in the surgery–pain–functioning pathway. The pain thresholds on postoperative day (POD) 1, 2, and 3 for optimal functional recovery were identified, corresponding to the cutoff points of pain categorization that demonstrated the largest indirect effects in the models.

**Results:**

The surgical approach had a significant indirect effect on activity limitation and walking difficulty through pain severity (*p* < 0.001), with a standardized effect value of 0.039 and 0.037, respectively. According to the largest mediation effects of pain categories generated from the each day SEM, the optimal pain score cutoffs are 5 on POD1, 4 on POD2, and 3 on POD3, for both activity limitation and walking difficulty.

**Conclusion:**

Our study quantified the partial mediating effects of pain between surgical approaches and postoperative functional status in patients with lung cancer surgery. The mediation effect–based pain thresholds support precise strategies for postoperative functional rehabilitation, which is considered the major goal of enhanced recovery after surgery.

**Trial Registration:** Chinese Clinical Trials Registry: ChiCTR2000033016

## 1. Introduction

Surgery is the primary treatment for lung cancer, but patients often experience significant and fluctuating symptom burdens during postoperative hospitalization [1]. Pain was the most frequently reported symptom among patients undergoing thoracic surgery, yet fewer than half experienced complete pain relief [2]. Video-assisted thoracoscopic surgery (VATS) has been employed in 80% of lung cancer surgeries [3, 4], characterized by a shorter recovery period and fewer postoperative complications. However, many patients still experience intense pain during the early stage after VATS [2, 5–8], which significantly impacts their daily functioning, such as walking and general activity.

Currently, enhanced recovery after surgery (ERAS) has been proven to reduce the occurrence of surgical complications and accelerate recovery [9, 10]. Postoperative dynamic pain management and functional recovery are both key elements of the ERAS protocol. Optimal pain management facilitates early functional recovery after surgery and then shortens the length of hospital stay [5, 11, 12]. Measured by patient-reported outcomes (PROs), symptoms and functional status [13] offer a direct and personalized perspective from patients, compared to traditional clinical outcomes [14, 15].

In clinical practice, it is necessary to guide pain management based on thresholds of severity scores. However, current clinical guidelines often provide pain thresholds that more based on chronic pain than acute pain associated with surgery, and these cutoff points vary across different clinical settings [16]. For example, the National Comprehensive Cancer Network (NCCN) Clinical Practice Guidelines in Oncology for Adult Cancer Pain suggest that a 0–10 numerical rating scale (NRS) of ≥ 4 indicates moderate to severe pain and requires certain interventions [17]. Currently, no specific thresholds is available for postoperative pain which changes dramatically over the acute phase after surgery, leading to corresponding changes in function and necessitating dynamic strategies for rehabilitation.

Additionally, promoting function is a crucial perioperative goal [16]. Our previous study compared the recovery trajectories of patients undergoing thoracotomy versus VATS, demonstrating significant differences in surgery approach–related symptom severity, which impacted their physical function and quality of life [18]. Merlo et al. identified pain as a core component of symptom clusters that impair quality of life following thoracic surgery [19], whereas Lakra et al. demonstrated that early postoperative pain predicts long-term functional outcomes after knee arthroplasty [20]. These findings collectively highlight the critical role of effective pain management in achieving optimal functional recovery. Thus, early and precise thresholds of pain based on functional recovery are required, to improve pain assessment and management, as well as overall prognosis for patients.

This study aims to investigate what extent pain mediates the association between the different surgical approaches (single-port VATS versus multiport VATS or thoracotomy) and postoperative functional recovery *via* the structural equation model (SEM) [21]. We also aim to identify the optimal pain score threshold for each hospitalization day after surgery, to provide clinicians with precise and practical pain management strategies, anchored via the mediation effects of pain between surgical approach and functional status.

## 2. Methods

### 2.1. Study Design and Participants

**Figure 1:**
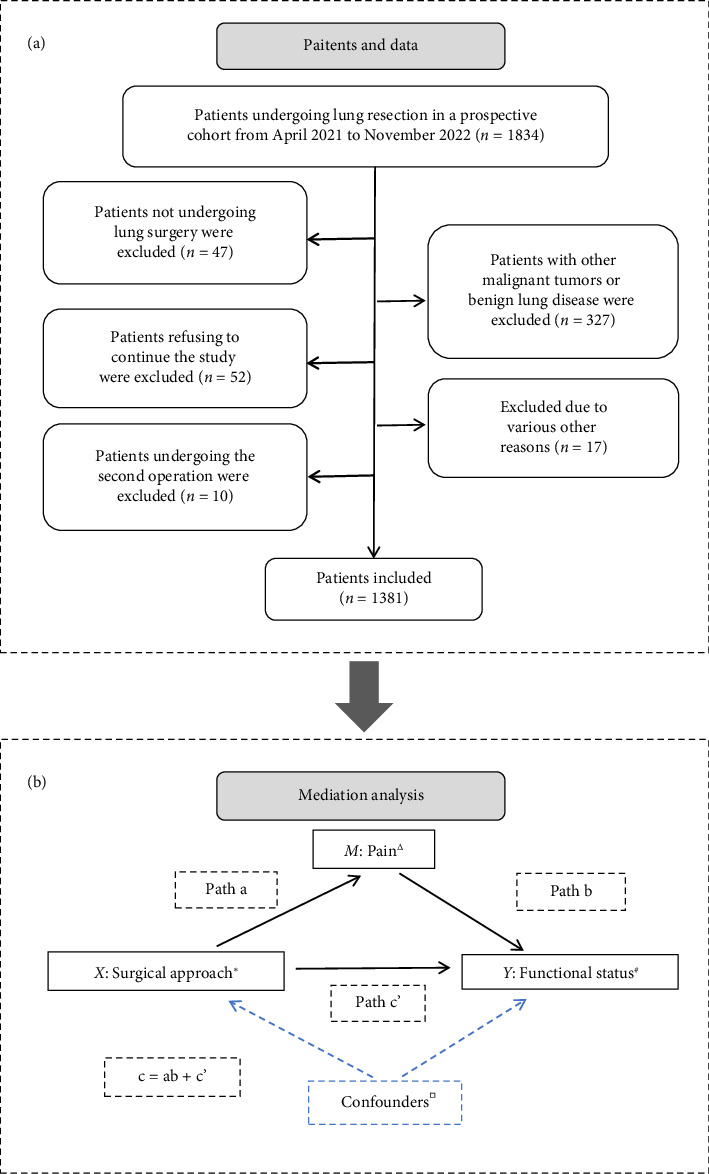
Flowchart of patient selection (a) and the directed acyclic graph representing relationship between surgical approach and functional status (b). ^∗^Surgical approach: single-port VATS (coded as 0); multiport VATS or thoracotomy (coded as 1). ^△^Pain was set as different variable types for different purposes of mediating analyses. As a continuous variable, a dataset of the first preoperative day to discharge was used for determining its mediating effect. As a dichotomous variable, a dataset of the first three postoperative days was used for identifying its optimal cutoff point. ^#^Functional status: activity limitation (*Y*1); walking difficulty (*Y*2). ^□^Confounders including demographic characteristics (age, gender, smoking status, drinking history, BMI, education level, and annual household income) and baseline clinical information (CCI score, histological type, pathological TNM stage, FEV1, FEV1%, ASA classification, and preoperative PRO scores). Control variable: Propensity score, postoperative time, and daily morphine equivalent doses were included in path a; propensity score and postoperative time were included in path b and path c'. The parameters of SEM were estimated with bootstrapping 1000 times. Abbreviations: SEM, structural equation model; VATS, video-assisted thoracoscopic surgery; BMI, body mass index; FEV1, forced expiratory volume in 1 s; CCI, Charlson Comorbidity Index; TNM, tumor-node-metastasis; ASA, American Society of Anesthesiologists.

This study utilized data extracted from an ongoing prospective cohort study (CN-PRO-Lung 3) conducted at the Sichuan Cancer Hospital, encompassing patients with lung cancer who underwent VATS or thoracotomy between April 7, 2021, and November 30, 2022. This study was registered with the Chinese Clinical Trials Registry and approved by the Ethics Committee of Sichuan Cancer Hospital (No. SCCHEC-02-2018-043). We adhered to the Strengthening the Reporting of Observational Studies in Epidemiology guideline. The inclusion criteria were as follows: aged ≥ 18 years, confirmed diagnosis of primary lung cancer, and with pathological stage I-IIIA according to the American Joint Committee on Cancer. The exclusion criteria were as follows: having undergone a second operation, not actually undergoing lung surgery, having other malignant tumors, or refusing to continue the study. All patients have signed informed consent forms. A flowchart of patient inclusion is shown in Figure 1(a).

### 2.2. Surgical Procedure

Surgical approaches were determined based on the preoperative evaluation and actual intraoperative findings. Thoracotomy was performed with a standard anterolateral or posterolateral incision. VATS included single-port VATS and multiport VATS. More details were described in our previous study [18]. Our study categorized the surgical approach into two groups: single-port VATS versus multiport VATS or thoracotomy approaches, between which significant differences in postoperative symptom burden were demonstrated in our previous analysis [8]. The multiport VATS or thoracotomy group includes two-port VATS, three-port VATS, four-port VATS, VATS converted to thoracotomy, and subxiphoid VATS. In our cohort, all patients received standardized care postoperatively, and chest drains were typically removed on postoperative day (POD) 2 or 3, depending on the patient's recovery progress, including factors such as the absence of air leaks, adequate lung expansion, and a drained fluid volume of < 200 mL/day [22].

### 2.3. Data Collection

Demographic characteristics included age, gender, smoking status, drinking history, body mass index (BMI), education level, and annual household income. Baseline clinical information included Charlson Comorbidity Index (CCI) score, histological type, pathological TNM stage, forced expiratory volume in one second (FEV1), ratio of forced expiratory volume in one second to the predicted value (FEV1%), American Society of Anesthesiologists (ASA) classification, and preoperative PRO scores. The PRO data were derived from the pain and two daily functional status (including activity limitation and walking difficulty) scores of the Perioperative Symptom Assessment for Lung Surgery (PSA-Lung) scale (Figure S1) [23]. The development team of the PSA-Lung scale previously confirmed its reliability and validity in patients undergoing lung cancer surgery. Each item is rated on a numerical scale from 0 to 10, with higher scores indicating worse symptoms or functional status. The recall period is 24 h. Symptom assessments were evaluated at baseline and each day during hospitalization, and postoperative morphine equivalent dosage [17] was daily recorded after surgery.

### 2.4. Statistical Analysis

#### 2.4.1. Descriptive Statistical Analysis

Descriptive statistics were used to summarize the demographic and clinical characteristics of the patients. Measurement data were presented as median and interquartile range (IQR) for nonnormally distributed variables and mean ± standard deviation for normally distributed variables. Measurement data were analyzed by the Mann–Whitney *U* test (nonnormal distribution) and independent *t* test (normal distribution). Categorical variables were compared using Pearson's chi-square test or Fisher's exact test and were presented as counts and percentages. These tests were used to identify imbalanced variables between the two subgroups.

#### 2.4.2. Control Variables

Base on the prior knowledge and clinical experience [8, 18], a directed acyclic graph (DAG) was constructed to identify potential pretreatment confounders (Figure 1(b)). The propensity score (PS) method was utilized to control for confounders and adjusted for those with inconsistent distributions between the two subgroups. First, a logistic regression probability model was established for the patients undergoing a specific surgical approach. Then, for each patient, the probability score of undergoing the specific surgical approach was calculated using the established PS model. The PS was included in the SEM as a control variable to help mitigate the impact of confounding factors. The SEM incorporated all available daily assessments from POD 1 until discharge for each patient. To account for the unbalanced number of observations per patient and to control for the confounding effect of the recovery timeline itself, postoperative time (modeled as a continuous variable representing the day of assessment relative to the surgery day) was included as a key control variable in the models. Additionally, daily morphine equivalent doses were also included as control variables.

#### 2.4.3. Causal Mediation Analysis

The statistical interpretation of mediation analysis can be broken down into separate effects. We estimated the surgical approach on pain (path a), the effect of pain on functional status (path b), and the indirect effect of surgical approach on functional status through pain (path ab). Path c' is the direct effect of surgical approach on functional status. Path *c* denotes the total effect, equal to direct effect plus indirect effect (c'+ab) (Figure 1(b)). SEM provides path coefficients (i.e., standardized effect values *β*) to evaluate effect strength, as well as the product of coefficients of the mediating effect with a bias-corrected bootstrapped confidence interval (CI), currently seen as an optimal method for mediation analysis [24].

The longitudinal analysis utilized data from POD 1 until the day of discharge to assess the mediation effect across the entire early recovery phase. In contrast, the analysis to identify the optimal pain thresholds was conducted separately for each of the first three PODs (POD 1 to POD 3). A longitudinal SEM (LSEM) was employed to investigate the relationship among surgical approach, functional status (activity limitation or walking difficulty), and pain. For this analysis, we utilized all available daily assessments from POD1 until discharge for both the mediating variable (pain scores) and outcome variables (functional status scores). The surgical approaches were selected as the independent variable, represented as a binary variable: single-port VATS (coded as 0) versus multiport VATS or thoracotomy (coded as 1). Longitudinally postoperative functional status scores were used as outcome variables, with postoperative pain serving as the mediating variable.

The first model assessed the overall impact of the surgical approach on activity limitation (*Y*1), adding pain as a mediator. The second model evaluated the overall impact of the surgical approach on walking difficulty (*Y*2), also incorporating pain as a mediator. Both models utilized the bootstrapping method with 1000 resamples to obtain parameter estimates for total and indirect effects. A 95% CI was used to determine the statistical significance of the effects: if the 95% CI did not include zero, the indirect effect was considered statistically significant, indicating the presence of a mediation effect.

#### 2.4.4. Optimal Thresholds of Pain Severity After Lung Cancer Surgery

To investigate the optimal threshold of pain score for clinical intervention, the pain scores were included in the SEM as a dichotomous variable. SEM with different cutoff points of pain scores was constructed on POD1, POD2, and POD3. The pain scores were dichotomized at nine different cutoff points, specifically at scores of 1, 2, 3, 4, 5, 6, 7, 8, and 9 [17]. Each cutoff point divided the pain scores into two groups: one group with scores less than the cutoff point and another group with scores equal or greater than or equal to the cutoff point. For each cutoff point, a SEM was constructed, using the dichotomized pain score as a mediator variable to estimate the relevant parameters. The optimal threshold for clinical intervention was determined by comparing the proportion of indirect effects in the models under different cutoff points. All models utilized the bootstrapping method with 1000 resamples to obtain parameter estimates for total and indirect effects.

#### 2.4.5. Sensitivity Analysis

The missing data were processed by the full information maximum likelihood (FIML) method within the SEM, which utilizes all available data for model parameter estimation. The first sensitivity analysis was conducted to assess the possible impact of missing data. The mediation effect was investigated using complete cases through listwise deletion to remove missing data. The second sensitivity analysis was used to evaluate the impact of the PS method on the mediation model. The mediation modeling and the optimal cutoff points were explored using a dataset without PS. The sensitivity analyses were performed to ensure the robustness of the model.

#### 2.4.6. Model Fit Evaluation

The fit indices were used to evaluate the fit of the constructed model to the existing data. Specific indices included *χ*^2^ statistic, *p* value, goodness-of-fit index (GFI), comparative fit index (CFI), Tucker–Lewis index (TLI), standardized root mean square residual (SRMR), and root mean square error of approximation (RMSEA). Good or adequate model fit is indicated by a nonsignificant *χ*^2^ value, GFI > 0.90, CFI > 0.90, TLI > 0.90, and SRMR and RMSEA ≤ 0.08 [25–28].

All statistical analyses were performed using *R* 4.3.3 (https://www.r-project.org/). The SEM was constructed using the lavaan package in R. Standardized regression coefficients for all hypothesized paths were estimated using the maximum likelihood method with the parameter estimates and covariance matrix from the lavaan package. A two-sided *p* value < 0.05 was considered statistically significant.

## 3. Results

### 3.1. Patient Characteristics and Intergroup Imbalance Variables

Of the 1834 patients in the longitudinal CN-PRO-Lung 3 study, 1381 were included in the analysis. There were intergroup imbalances in BMI (*p* < 0.001), education level (*p* < 0.001), annual household income (*p*=0.003), smoking history (*p* < 0.001), drinking history (*p*=0.016), pathological type (*p* < 0.001), TNM stage (*p* < 0.001), CCI (*p*=0.006), and preoperative walking difficulty score (*p*=0.013) between the two groups (Table 1). These intergroup imbalances were adjusted by the PS method to mitigate the bias.

### 3.2. Mediation Analysis

A mediation analysis evaluated the mediating effect of pain on the relationship between surgical approach and functional status using SEM (Figure 1(b)). The surgical approach exerted a significant indirect mediating effect on activity limitation through the pain score (*p* < 0.001); meanwhile, for the walking difficulty, similar results were obtained (Table 2 and Figure S2). Path a in this model is interpreted as an average treatment effect because of the treatment allocation variable being binary (single-port VATS = 0). Taking the LSEM with activity limitation (*Y*1) as example, the value of 0.072 for path a between surgical approach and pain therefore can be interpreted as the change in pain being 0.072 units higher in the multiport VATS or thoracotomy group than in the single-port VATS group. Similarly, path *b* of 0.534 can be interpreted as that a 1 unit change in pain leads to a 0.534 units change in activity limitation (Figure S1). When the mediator (pain) was added to the model, pathway ab explained 54.17% proportion of the treatment effect, and a statistically significant mediating effect was found (*β* = 0.039, Table 2). The fit indices of each model were excellent (Table 3).

### 3.3. Optimal Threshold of Pain Severity for Each Day After Lung Surgery

**Figure 2:**
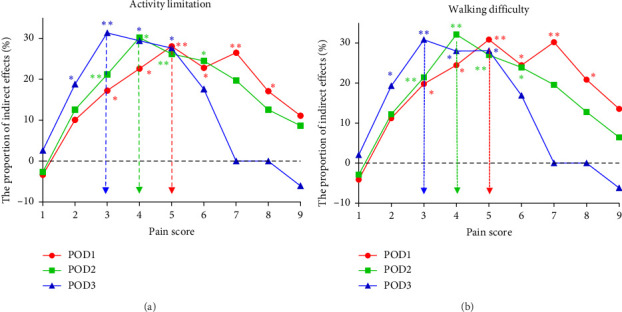
The proportion of indirect effects of activity limitation (a) and walking difficulty (b) on POD1 to POD3 dichotomized by different cutoff points of pain scores. Activity limitation as the outcome, the optimal cutoffs were 5 on POD1, 4 on POD 2, and 3 on POD3, with the highest proportion of indirect effect being 28.07%, 30.20%, and 31.33%, respectively. Walking difficulty as the outcome, similar results were obtained, with the highest proportion of indirect effect being 30.85%, 32.12%, and 30.82%, respectively. Abbreviation: POD, postoperative day. ∗: *p* < 0.05; ∗∗: *p* < 0.01.

In the SEM with activity limitation (*Y*1) as the outcome, the optimal cutoffs were 5 on POD1, 4 on POD 2, and 3 on POD3, with the highest proportion of indirect effect being 28.07%, 30.20%, and 31.33%, respectively (Figure 2(a) and Tables S1–S3). In the SEM with walking difficulty (*Y*2) as the outcome, similar results were obtained, with the highest proportion of indirect effect being 30.85%, 32.12%, and 30.82%, respectively (Figure 2(b) and Tables S4–S6).

**Figure 3:**
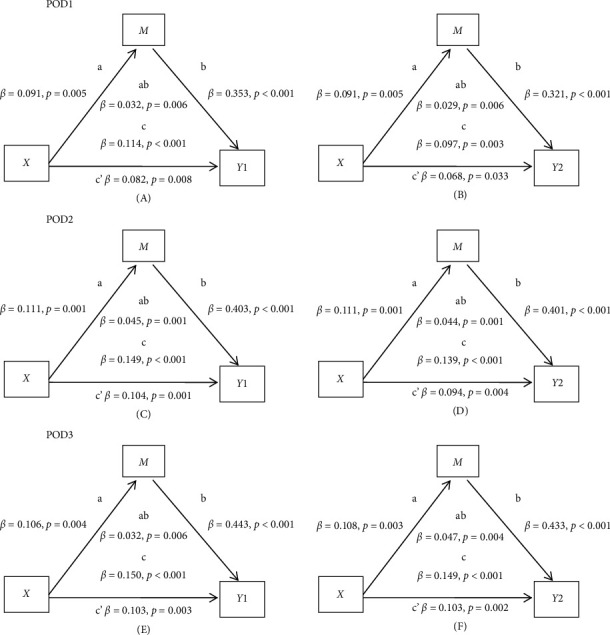
Mediation effects at the optimal cutoff point of pain scores *via* SEM on POD1 (A/B), POD2 (C/D), and POD3 (E/F). *X*: Surgical approach (single-port VATS versus multiport VATS or thoracotomy). *M*: Pain (dichotomized at the optimal cutoff point). *Y*: Functional status (activity limitation [*Y*1]; walking difficulty [*Y*2]). Mediated effects are the product of a and b coefficients (ab). All values are standardized. Abbreviation: SEM, structural equation model.

Based on the above results, a pathway diagram of SEM was used to demonstrate how surgical approach affects daily function status (including activity limitation and walking difficulty) mediated by pain scores, using cutoff score of 5 on POD1, 4 on POD2, and 3 on POD3 (Figure 3). The fit indices of each model were excellent (Table S7).

### 3.4. Sensitivity Analysis

The first sensitivity analysis demonstrated that pain was also a significant mediating factor between the surgical approach and functional status after deleting the missing data imputation (*p* < 0.001, Table S8). In the second sensitivity analysis, the mediating effect of pain was significant without adopting PS as a control variable (Table S9). Similarly, the fit indices of SEM with different cutoff points of pain scores constructed on POD1, POD2, and POD3 were similar to the aforementioned results (Tables S10–S15). All the results were also not materially different in sensitivity analyses, increasing confidence in the observed associations.

## 4. Discussion

Our mediation analysis based on PRO quantified the mediation of pain in the relationship between the surgical approaches and postoperative daily functioning in patients with lung cancer surgery. Compared with patients who underwent thoracotomy or multiport VATS, patients who underwent single-port VATS had lower pain sores, partially leading to better daily function recovery. We firstly identified optimal pain score thresholds for functional recovery on each day from POD1 to POD3, requiring PRO-based scores below 5 on POD1, 4 on POD2, and 3 on POD3, providing evidence for postoperative pain management.

Pain is one of the most common and longest-lasting symptoms after lung cancer surgery and is associated with reduced physical function levels [29–33]. In our cohort, pain was the most severe symptom during the acute postoperative phase (POD1–3), with mean scores significantly higher than other symptoms (Table S16). Studies have shown that pain is the symptom that most commonly differs in severity between patients undergoing thoracotomy versus those undergoing VATS [2, 5–8]. Our study highlights the crucial role of pain management in postoperative functional recovery for thoracic surgery. However, the recommendations of guidelines for postoperative pain management are often too broad and generalized, lacking specificity to particular surgeries. Thus, healthcare providers often rely on standardized medication dosages and administration frequencies, without fully considering the patient's unique condition and needs, making it difficult to achieve individualized pain management [7, 19, 34].

Our study provides a refined early alert management of the daily optimal threshold for pain of patients after lung cancer surgery. We quantify the impact of pain as a mediator on functional recovery at different pain score cutoffs for the first 3 days postoperatively. The optimal cutoff point showed a decrease trend over time, 5 on POD1, 4 on POD2, and 3 on POD3. These findings indicate that clinical staff should closely monitor patients' pain and maintain their pain scores below 5 on POD 1, below 4 on POD 2, and below 3 on POD 3. That provides a reference for pain management with significant clinical implications, to promote postoperative functional recovery, potentially informing discharge decision making.

Our study focuses on physical functioning as the primary outcome, which aligns with the crucial goal of ERAS. Importantly, this concern is not limited to lung cancer surgeries but is relevant to all types of surgical procedures that prioritize functional recovery. Although VATS offers several advantages, postoperative pain remains a significant barrier to optimal recovery for VATS patients. Pain management is especially important for patients, as they face higher degrees of physical trauma and a greater risk of complications, thus requiring more meticulous management plans to ensure rapid functional recovery [35].

ERAS recommends early management of postoperative pain through a series of clinically validated effective measures, including multimodal analgesia [36, 37]. Most guidelines adopt a one-size-fits-all approach that pain requires clinical attention when rated ≥ NRS 4 (in a 0–10 scale) [17]. Unlike chronic pain associated with conditions like cancer and orthopedic issues, surgical pain is acute and changes rapidly. Therefore, the approach to postoperative analgesia should be “as needed,” with a prerequisite for accurate and timely pain assessment. Our study meticulously assessed the functional status on each day postsurgery, enabling the determination of daily pain threshold. Early postoperative analgesia is key to functional recovery, so we advocate for precisely controlling pain levels below threshold during the first 3 days, with specific pain thresholds provided for each day to enhance pain management precision. Our findings are of significant practical importance.

This study has several limitations. First, although we considered the overall impact (from the first preoperative day to discharge) of the surgical approach on daily function recovery when inspected the mediating effect of pain, only the data of the first three PODs were included when dichotomizing the pain score to identify the optimal threshold. Additionally, the intensity of pain is higher during this period, with the greatest need for pain management. It is known that thoracic surgery is associated with chronic postsurgical pain [38], so long-term postoperative and postdischarge situations warrant further research. Second, the identified indirect effects, though small, are statistically robust and consistent, suggesting meaningful population-level clinical relevance. Mediation analysis highlights pain severity's unique role as a mediator, distinct from other mechanisms. Consistency across functional outcomes reinforces its robustness. Despite modest effect sizes, pain thresholds derived from maximizing mediation proportion provide precise, clinically actionable targets. Finally, to account for the significant impact of analgesic management on postoperative pain, which is itself influenced by the initial anesthetic methods and techniques, the daily morphine equivalent dose of opioid analgesics was included as a control variable in the models. The use of nonsteroidal anti-inflammatory drugs (NSAIDs) was not considered. Nonetheless, 100% of the patients included in this study used NSAIDs, and there was no difference between the groups. Additionally, while our study focused on pain as the critical mediator between surgical approach and functional recovery, other postoperative symptoms may independently or synergistically influence outcomes. However, we prioritized pain due to its high prevalence, acute dynamicity in the early postoperative phase, and established direct association with physical limitations in thoracic surgery. Future studies should extend this mediation framework to incorporate multimodal symptom interactions, providing a more comprehensive understanding of recovery pathways.

## 5. Conclusion

Our findings suggested that pain partially mediated the relationship between different surgical approaches and postoperative daily function recovery in patients after lung cancer surgery. We also provided the optimal threshold reference for postoperative thoracic surgery patients, emphasizing the importance of closely monitoring the pain severity of patients during the first 3 days after surgery. This strategy supports the implementation of the ERAS concept for postoperative lung cancer patients, ensuring a swift and effective recovery to a healthy state.

## Figures and Tables

**Table 1:** Comparison of baseline demographic and patient characteristics between single-port VATS and multiport VATS/thoracotomy groups in preoperative patients with lung cancer.

	Single-port VATS (*N* = 862)	Multiport VATS or thoracotomy (*N* = 519)	Total (*N* = 1381)	*p* value
*Age*				
< 60	591 (68.6%)	329 (63.4%)	920 (66.6%)	0.148^b^
≥ 60	271 (31.4%)	190 (36.6%)	461 (33.4%)	
BMI, IQR	22.4 (15.4, 38.8)	23.1 (14.5, 36.1)	22.7 (14.5, 38.8)	< 0.001^a^

*Gender*				
Male	303 (35.2%)	210 (40.5%)	513 (37.1%)	0.122^b^
Female	559 (64.8%)	309 (59.5%)	868 (62.9%)	
Education level				
Middle school graduate or below	515 (59.7%)	371 (71.5%)	886 (64.2%)	< 0.001^b^
Above middle school graduate	347 (40.3%)	148 (28.5%)	495 (35.8%)	

*Annual household income*				
< 100,000 CNY	567 (65.8%)	386 (74.4%)	953 (69.0%)	0.003^b^
≥ 100,000 CNY	295 (34.2%)	133 (25.6%)	428 (31.0%)	

*Smoking status*				
Never	697 (80.9%)	369 (71.1%)	1066 (77.2%)	< 0.001^b^
Current or former	165 (19.1%)	150 (28.9%)	315 (22.8%)	

*Drinking history*				
No	744 (86.3%)	417 (80.3%)	1161 (84.1%)	0.016^b^
Yes	118 (13.7%)	102 (19.7%)	220 (15.9%)	

FEV1 (*L*)^∗^				
< 1.5	153 (17.7%)	86 (16.6%)	239 (17.3%)	0.853^b^
≥ 1.5	679 (78.8%)	416 (80.2%)	1095 (79.3%)	

*FEV*1%⁣(*measured*/*predicted* %)^#^				
< 60%	123 (14.3%)	63 (12.1%)	186 (13.5%)	0.505^b^
≥ 60%	701 (81.3%)	436 (84.0%)	1137 (82.3%)	

*Histological type*				
Adenocarcinoma	817 (94.8%)	455 (87.7%)	1272 (92.1%)	< 0.001^b^
Nonadenocarcinoma	45 (5.2%)	64 (12.3%)	109 (7.9%)	

*TNM stage*				
Early (stage 0-I)	797 (92.5%)	435 (83.8%)	1232 (89.2%)	< 0.001^b^
Advanced (stage II–IV)	65 (7.5%)	84 (16.2%)	149 (10.8%)	

*CCI score*				
0	253 (29.4%)	112 (21.6%)	365 (26.4%)	0.006^b^
≥ 1	609 (70.6%)	407 (78.4%)	1016 (73.6%)	

*ASA classification*				
≤ 1	783 (90.8%)	470 (90.6%)	1253 (90.7%)	0.982^b^
> 1	79 (9.2%)	49 (9.4%)	128 (9.3%)	

*Preoperative PRO*				
Pain, IQR	0 (0, 6)	0 (0, 10)	0 (0, 10)	0.473^a^
Activity limitation, IQR	0 (0, 8)	0 (0, 8)	0 (0, 8)	0.372^a^
Walking difficulty, IQR	0 (0, 7)	0 (0, 10)	0 (0, 10)	0.013^a^

Abbreviations: ASA, American Society of Anesthesiologists; BMI, body mass index; CCI, Charlson Comorbidity Index; FEV1, forced expiratory volume in 1 s; TNM, tumor-node-metastasis; VATS, video-assisted thoracoscopic surgery.

^a^Mann–Whitney *U* test.

^b^Chi-square tests.

^∗^FEV1 missing 47 (3.4%) in total, with 30 (3.5%) vs. 17 (3.3%).

^#^FEV1% missing 58 (4.2%) in total, with 38 (4.4%) vs. 20 (3.9%).

**Table 2:** Path analysis of mediating effects of pain between surgical approach and activity limitation/walking difficulty.

Variable	Path	Effect	Result			*Z*	*p*	95% CI		Proportion of total effect
			*β*	Estimate	SE			Lower	Upper	
*Y*1	ab	indirect effect	0.039	0.204	0.048	4.261	< 0.001	0.116	0.304	54.17%
	c'	direct effect	0.033	0.176	0.076	2.319	0.020	0.024	0.318	45.83%
	c	total effect	0.072	0.379	0.090	4.238	< 0.001	0.211	0.555	100.00%

*Y*2	ab	indirect effect	0.037	0.197	0.046	4.301	< 0.001	0.112	0.291	56.06%
	c'	direct effect	0.029	0.157	0.077	2.028	0.043	0.002	0.310	43.94%
	c	total effect	0.066	0.354	0.090	3.943	< 0.001	0.182	0.532	100.00%

*Note: Y*1, activity limitation; *Y*2, walking difficulty. *β*, standardized effect value. Path *ab*: path *a* and *b* make up the mediating pathway, also being described as the indirect effect. Path *c'*: the direct effect of treatment on outcome. Path *c*: the total effect, equal to direct effect plus indirect effect (*c'* + *a* ∗ *b*).

**Table 3:** Fit index based on two mediation effect models of activity limitation and walking difficulty.

Fit index	*χ* ^2^	*p* value	GFI	CFI	TLI	SRMR	RMSEA
*Y*1	0.006	0.938	0.999	1.000	1.000	0.000	0.000
*Y*2	0.502	0.478	0.999	1.000	1.000	0.002	0.000
Standard	—	> 0.05	> 0.90	> 0.90	> 0.90	< 0.08	< 0.08

*Note: Y*1, activity limitation; *Y*2, walking difficulty; standard, thresholds of good model fit.

Abbreviations: CFI, comparative fit index; GFI, goodness-of-fit index; RMSEA, root mean square error of approximation; SRMR, standardized root mean square residual; TLI, Tucker–Lewis index.

## Data Availability

The data that support the findings of this study are available from the corresponding author upon reasonable request.

## Supporting Information

Supporting data include 16 tables and 2 figures.

Table S1. Path analysis of mediating effects of pain between surgical approach and activity limitation on POD1 dichotomized by different cutoff points of pain scores.

Table S2. Path analysis of mediating effects of pain between surgical approach and activity limitation on POD2 dichotomized by different cutoff points of pain scores.

Table S3. Path analysis of mediating effects of pain between surgical approach and activity limitation on POD3 dichotomized by different cutoff points of pain scores.

Table S4. Path analysis of mediating effects of pain between surgical approach and walking difficulty on POD1 dichotomized by different cutoff points of pain scores.

Table S5. Path analysis of mediating effects of pain between surgical approach and walking difficulty on POD2 dichotomized by different cutoff points of pain scores.

Table S6. Path analysis of mediating effects of pain between surgical approach and walking difficulty on POD3 dichotomized by different cutoff points of pain scores.

Table S7. Fit index based on two mediation effect models of activity limitation and walking difficulty on POD1 to POD3.

Table S8. Path analysis of mediating effects of pain between surgical approach and activity limitation/walking difficulty without missing data imputation.

Table S9. Path analysis of mediating effects of pain between surgical approach and activity limitation/walking difficulty without propensity score.

Table S10. Path analysis of mediating effects of pain between surgical approach and activity limitation on POD1 without consideration of propensity scores using different cutoff points of pain scores.

Table S11. Path analysis of mediating effects of pain between surgical approach and activity limitation on POD2 without consideration of propensity scores dichotomized by different cutoff points of pain scores.

Table S12. Path analysis of mediating effects of pain between surgical approach and activity limitation on POD3 without consideration of propensity scores dichotomized by different cutoff points of pain scores.

Table S13. Path analysis of mediating effects of pain between surgical approach and walking difficulty on POD1 without consideration of propensity scores dichotomized by different cutoff points of pain scores.

Table S14. Path analysis of mediating effects of pain between surgical approach and walking difficulty on POD2 without consideration of propensity scores dichotomized by different cutoff points of pain scores.

Table S15. Path analysis of mediating effects of pain between surgical approach and walking difficulty on POD3 without consideration of propensity scores dichotomized by different cutoff points of pain scores.

Table S16 Patient symptom scores from postoperative day 1 to day 3.

Figure S1. Perioperative Symptom Assessment-Lung Surgery (PSA-Lung).

Figure S2. Mediation effects identified by the LSEM.
