# Supplementary material for: Establishing Pain Thresholds for Functional Recovery After Lung Cancer Surgery: A Mediation Analysis of the Surgery–Pain–Functioning Causal Pathway
Source: Pain Res Manag. 2025 Nov 28;2025:3783225. doi: 10.1155/prm/3783225 (PMC12680459; doi:10.1155/prm/3783225)
Supplement: Supporting Information — Additional supporting information can be found online in the Supporting Information section. [file 3783225.f1.docx]

# Supplementary Data

# Table S1. Path analysis of mediating effects of pain between surgical approach and activity limitation on POD1 dichotomized by different cut-off points of pain scores.

| M | Path | Effect | Result | | | Z | *P* | 95% CI | | Proportion of total effect |
| --- | --- | --- | --- | --- | --- | --- | --- | --- | --- | --- |
|  |  |  | *β* | Estimate | SE |  |  | lower | upper |  |
| 1 | ab | indirect effect | -0.004 | -0.022 | 0.045 | -0.503 | 0.615 | -0.116 | 0.061 | -3.36% |
|  | c’ | direct effect | 0.123 | 0.770 | 0.195 | 3.945 | <0.001 | 0.384 | 1.17 | 103.36% |
|  | c | total effect | 0.119 | 0.747 | 0.200 | 3.738 | <0.001 | 0.358 | 1.148 | 100.00% |
| 2 | ab | indirect effect | 0.012 | 0.074 | 0.049 | 1.505 | 0.132 | -0.027 | 0.165 | 10.08% |
|  | c’ | direct effect | 0.107 | 0.670 | 0.196 | 3.422 | 0.001 | 0.281 | 1.064 | 89.92% |
|  | c | total effect | 0.119 | 0.743 | 0.200 | 3.717 | <0.001 | 0.350 | 1.146 | 100.00% |
| 3 | ab | indirect effect | 0.020 | 0.126 | 0.061 | 2.070 | 0.038 | 0.005 | 0.238 | 17.39% |
|  | c’ | direct effect | 0.095 | 0.597 | 0.190 | 3.133 | 0.002 | 0.24 | 0.980 | 82.61% |
|  | c | total effect | 0.115 | 0.722 | 0.200 | 3.605 | <0.001 | 0.329 | 1.130 | 100.00% |
| 4 | ab | indirect effect | 0.026 | 0.162 | 0.066 | 2.458 | 0.014 | 0.025 | 0.292 | 22.61% |
|  | c’ | direct effect | 0.089 | 0.555 | 0.192 | 2.892 | 0.004 | 0.186 | 0.940 | 77.39% |
|  | c | total effect | 0.115 | 0.717 | 0.200 | 3.584 | <0.001 | 0.325 | 1.124 | 100.00% |
| **5** | **ab** | **indirect effect** | **0.032** | **0.201** | **0.073** | **2.750** | **0.006** | **0.055** | **0.340** | **28.07%** |
|  | c’ | direct effect | 0.082 | 0.513 | 0.194 | 2.640 | 0.008 | 0.143 | 0.898 | 71.93% |
|  | c | total effect | 0.114 | 0.714 | 0.200 | 3.568 | <0.001 | 0.321 | 1.122 | 100.00% |
| 6 | ab | indirect effect | 0.026 | 0.161 | 0.076 | 2.101 | 0.036 | 0.011 | 0.306 | 22.81% |
|  | c’ | direct effect | 0.088 | 0.551 | 0.194 | 2.833 | 0.005 | 0.184 | 0.938 | 77.19% |
|  | c | total effect | 0.114 | 0.711 | 0.200 | 3.559 | <0.001 | 0.324 | 1.118 | 100.00% |
| 7 | ab | indirect effect | 0.031 | 0.195 | 0.071 | 2.744 | 0.006 | 0.048 | 0.335 | 26.50% |
|  | c’ | direct effect | 0.086 | 0.538 | 0.194 | 2.768 | 0.006 | 0.157 | 0.932 | 73.50% |
|  | c | total effect | 0.117 | 0.733 | 0.200 | 3.672 | <0.001 | 0.342 | 1.138 | 100.00% |
| 8 | ab | indirect effect | 0.020 | 0.126 | 0.061 | 2.069 | 0.039 | 0.010 | 0.253 | 17.09% |
|  | c’ | direct effect | 0.097 | 0.608 | 0.195 | 3.118 | 0.002 | 0.240 | 1.005 | 82.91% |
|  | c | total effect | 0.117 | 0.733 | 0.199 | 3.678 | <0.001 | 0.347 | 1.141 | 100.00% |
| 9 | ab | indirect effect | 0.013 | 0.082 | 0.051 | 1.617 | 0.106 | -0.008 | 0.194 | 11.11% |
|  | c’ | direct effect | 0.104 | 0.652 | 0.196 | 3.333 | 0.001 | 0.284 | 1.040 | 88.89% |
|  | c | total effect | 0.117 | 0.734 | 0.200 | 3.677 | <0.001 | 0.355 | 1.136 | 100.00% |

Abbreviations: POD, postoperative day

**Table S2. Path analysis of mediating effects of pain between surgical approach and activity limitation on POD2 dichotomized by different cut-off points of pain scores.**

| M | Path | Effect | Result | | | Z | *P* | 95% CI | | Proportion of total effect |
| --- | --- | --- | --- | --- | --- | --- | --- | --- | --- | --- |
|  |  |  | *β* | Estimate | SE |  |  | lower | upper |  |
| 1 | ab | indirect effect | -0.004 | -0.02 | 0.032 | -0.638 | 0.523 | -0.088 | 0.031 | -2.65% |
|  | c’ | direct effect | 0.155 | 0.795 | 0.174 | 4.580 | <0.001 | 0.421 | 1.132 | 102.65% |
|  | c | total effect | 0.151 | 0.775 | 0.177 | 4.376 | <0.001 | 0.395 | 1.112 | 100.00% |
| 2 | ab | indirect effect | 0.019 | 0.097 | 0.048 | 2.003 | 0.045 | -0.006 | 0.19 | 12.58% |
|  | c’ | direct effect | 0.132 | 0.674 | 0.169 | 3.994 | <0.001 | 0.320 | 1.002 | 87.42% |
|  | c | total effect | 0.151 | 0.771 | 0.177 | 4.358 | <0.001 | 0.400 | 1.112 | 100.00% |
| 3 | ab | indirect effect | 0.032 | 0.165 | 0.056 | 2.923 | 0.003 | 0.048 | 0.266 | 21.33% |
|  | c’ | direct effect | 0.118 | 0.605 | 0.166 | 3.639 | <0.001 | 0.241 | 0.927 | 78.67% |
|  | c | total effect | 0.150 | 0.770 | 0.177 | 4.349 | <0.001 | 0.399 | 1.113 | 100.00% |
| **4** | **ab** | **indirect effect** | **0.045** | **0.228** | **0.070** | **3.250** | **0.001** | **0.092** | **0.365** | **30.20%** |
|  | c’ | direct effect | 0.104 | 0.534 | 0.161 | 3.309 | 0.001 | 0.192 | 0.836 | 69.80% |
|  | c | total effect | 0.149 | 0.762 | 0.177 | 4.297 | <0.001 | 0.387 | 1.111 | 100.00% |
| 5 | ab | indirect effect | 0.039 | 0.198 | 0.072 | 2.749 | 0.006 | 0.055 | 0.34 | 26.17% |
|  | c’ | direct effect | 0.110 | 0.562 | 0.158 | 3.555 | <0.001 | 0.226 | 0.856 | 73.83% |
|  | c | total effect | 0.149 | 0.760 | 0.177 | 4.292 | <0.001 | 0.387 | 1.111 | 100.00% |
| 6 | ab | indirect effect | 0.037 | 0.188 | 0.078 | 2.398 | 0.016 | 0.045 | 0.345 | 24.50% |
|  | c’ | direct effect | 0.114 | 0.582 | 0.158 | 3.694 | <0.001 | 0.254 | 0.886 | 75.50% |
|  | c | total effect | 0.151 | 0.770 | 0.178 | 4.336 | <0.001 | 0.395 | 1.116 | 100.00% |
| 7 | ab | indirect effect | 0.029 | 0.147 | 0.077 | 1.915 | 0.056 | -0.002 | 0.303 | 19.73% |
|  | c’ | direct effect | 0.118 | 0.605 | 0.162 | 3.724 | <0.001 | 0.265 | 0.911 | 80.27% |
|  | c | total effect | 0.147 | 0.752 | 0.178 | 4.223 | <0.001 | 0.376 | 1.097 | 100.00% |
| 8 | ab | indirect effect | 0.019 | 0.099 | 0.060 | 1.635 | 0.102 | -0.021 | 0.223 | 12.67% |
|  | c’ | direct effect | 0.131 | 0.672 | 0.167 | 4.016 | <0.001 | 0.322 | 1.001 | 87.33% |
|  | c | total effect | 0.150 | 0.771 | 0.178 | 4.338 | <0.001 | 0.389 | 1.113 | 100.00% |
| 9 | ab | indirect effect | 0.013 | 0.065 | 0.051 | 1.282 | 0.200 | -0.037 | 0.166 | 8.67% |
|  | c’ | direct effect | 0.137 | 0.700 | 0.17 | 4.105 | <0.001 | 0.365 | 1.039 | 91.33% |
|  | c | total effect | 0.150 | 0.765 | 0.178 | 4.307 | <0.001 | 0.382 | 1.107 | 100.00% |

Abbreviations: POD, postoperative day

**Table S3. Path analysis of mediating effects of pain between surgical approach and activity limitation on POD3 dichotomized by different cut-off points of pain scores.**

| M | Path | Effect | Result | | | Z | *P* | 95% CI | | Proportion of total effect |
| --- | --- | --- | --- | --- | --- | --- | --- | --- | --- | --- |
|  |  |  | *β* | Estimate | SE |  |  | lower | upper |  |
| 1 | ab | indirect effect | 0.004 | 0.018 | 0.032 | 0.549 | 0.583 | -0.053 | 0.073 | 2.63% |
|  | c’ | direct effect | 0.148 | 0.683 | 0.176 | 3.876 | <0.001 | 0.328 | 1.019 | 97.37% |
|  | c | total effect | 0.152 | 0.701 | 0.179 | 3.913 | <0.001 | 0.338 | 1.025 | 100.00% |
| 2 | ab | indirect effect | 0.028 | 0.128 | 0.057 | 2.234 | 0.025 | 0.003 | 0.239 | 18.79% |
|  | c’ | direct effect | 0.121 | 0.561 | 0.169 | 3.321 | 0.001 | 0.228 | 0.893 | 81.21% |
|  | c | total effect | 0.149 | 0.689 | 0.179 | 3.858 | <0.001 | 0.319 | 1.007 | 100.00% |
| **3** | **ab** | **indirect effect** | **0.047** | **0.217** | **0.075** | **2.868** | **0.004** | **0.068** | **0.361** | **31.33%** |
|  | c’ | direct effect | 0.103 | 0.476 | 0.16 | 2.967 | 0.003 | 0.16 | 0.791 | 68.67% |
|  | c | total effect | 0.150 | 0.692 | 0.178 | 3.879 | <0.001 | 0.325 | 1.005 | 100.00% |
| 4 | ab | indirect effect | 0.045 | 0.208 | 0.09 | 2.322 | 0.020 | 0.028 | 0.395 | 29.41% |
|  | c’ | direct effect | 0.108 | 0.498 | 0.154 | 3.234 | 0.001 | 0.200 | 0.795 | 70.59% |
|  | c | total effect | 0.153 | 0.706 | 0.179 | 3.953 | <0.001 | 0.34 | 1.017 | 100.00% |
| 5 | ab | indirect effect | 0.042 | 0.195 | 0.083 | 2.357 | 0.018 | 0.033 | 0.364 | 27.63% |
|  | c’ | direct effect | 0.110 | 0.506 | 0.155 | 3.274 | 0.001 | 0.204 | 0.799 | 72.37% |
|  | c | total effect | 0.152 | 0.701 | 0.178 | 3.927 | <0.001 | 0.338 | 1.010 | 100.00% |
| 6 | ab | indirect effect | 0.026 | 0.119 | 0.074 | 1.606 | 0.108 | -0.024 | 0.260 | 17.57% |
|  | c’ | direct effect | 0.122 | 0.564 | 0.16 | 3.519 | <0.001 | 0.245 | 0.865 | 82.43% |
|  | c | total effect | 0.148 | 0.683 | 0.179 | 3.82 | <0.001 | 0.318 | 0.997 | 100.00% |
| 7 | ab | indirect effect | 0 | -0.001 | 0.06 | -0.016 | 0.988 | -0.117 | 0.126 | 0.00% |
|  | c’ | direct effect | 0.146 | 0.672 | 0.166 | 4.048 | <0.001 | 0.328 | 0.977 | 100.00% |
|  | c | total effect | 0.146 | 0.671 | 0.179 | 3.757 | <0.001 | 0.305 | 0.986 | 100.00% |
| 8 | ab | indirect effect | 0 | -0.001 | 0.049 | -0.013 | 0.990 | -0.093 | 0.107 | 0.00% |
|  | c’ | direct effect | 0.151 | 0.696 | 0.171 | 4.081 | <0.001 | 0.348 | 1.014 | 100.00% |
|  | c | total effect | 0.151 | 0.696 | 0.179 | 3.882 | <0.001 | 0.322 | 1.013 | 100.00% |
| 9 | ab | indirect effect | -0.009 | -0.041 | 0.027 | -1.497 | 0.134 | -0.101 | 0.01 | -6.04% |
|  | c’ | direct effect | 0.158 | 0.728 | 0.177 | 4.103 | <0.001 | 0.355 | 1.046 | 106.04% |
|  | c | total effect | 0.149 | 0.687 | 0.179 | 3.845 | <0.001 | 0.316 | 0.997 | 100.00% |

Abbreviations: POD, postoperative day

**Table S4. Path analysis of mediating effects of pain between surgical approach and walking difficulty on POD1 dichotomized by different cut-off points of pain scores.**

| M | Path | Effect | Result | | | Z | *P* | 95% CI | | Proportion of total effect |
| --- | --- | --- | --- | --- | --- | --- | --- | --- | --- | --- |
|  |  |  | *β* | Estimate | SE |  |  | lower | upper |  |
| 1 | ab | indirect effect | -0.004 | -0.024 | 0.047 | -0.512 | 0.609 | -0.122 | 0.061 | -4.12% |
|  | c’ | direct effect | 0.101 | 0.646 | 0.202 | 3.203 | 0.001 | 0.237 | 1.032 | 104.12% |
|  | c | total effect | 0.097 | 0.622 | 0.208 | 2.986 | 0.003 | 0.196 | 1.024 | 100.00% |
| 2 | ab | indirect effect | 0.011 | 0.07 | 0.049 | 1.428 | 0.153 | -0.033 | 0.158 | 11.22% |
|  | c’ | direct effect | 0.087 | 0.555 | 0.203 | 2.730 | 0.006 | 0.143 | 0.954 | 88.78% |
|  | c | total effect | 0.098 | 0.625 | 0.209 | 2.998 | 0.003 | 0.198 | 1.033 | 100.00% |
| 3 | ab | indirect effect | 0.019 | 0.119 | 0.060 | 1.985 | 0.047 | 0.001 | 0.235 | 19.79% |
|  | c’ | direct effect | 0.077 | 0.492 | 0.199 | 2.478 | 0.013 | 0.093 | 0.888 | 80.21% |
|  | c | total effect | 0.096 | 0.611 | 0.209 | 2.930 | 0.003 | 0.181 | 1.014 | 100.00% |
| 4 | ab | indirect effect | 0.023 | 0.147 | 0.062 | 2.362 | 0.018 | 0.020 | 0.264 | 24.47% |
|  | c’ | direct effect | 0.071 | 0.454 | 0.202 | 2.244 | 0.025 | 0.047 | 0.871 | 75.53% |
|  | c | total effect | 0.094 | 0.601 | 0.209 | 2.880 | 0.004 | 0.172 | 1.005 | 100.00% |
| **5** | **ab** | **indirect effect** | **0.029** | **0.183** | **0.069** | **2.669** | **0.008** | **0.048** | **0.311** | **30.85%** |
|  | c’ | direct effect | 0.065 | 0.416 | 0.204 | 2.041 | 0.041 | -0.004 | 0.810 | 69.15% |
|  | c | total effect | 0.094 | 0.599 | 0.209 | 2.870 | 0.004 | 0.166 | 1.010 | 100.00% |
| 6 | ab | indirect effect | 0.023 | 0.147 | 0.071 | 2.057 | 0.040 | 0.011 | 0.286 | 24.47% |
|  | c’ | direct effect | 0.071 | 0.457 | 0.209 | 2.189 | 0.029 | 0.039 | 0.873 | 75.53% |
|  | c | total effect | 0.094 | 0.603 | 0.209 | 2.892 | 0.004 | 0.168 | 1.005 | 100.00% |
| 7 | ab | indirect effect | 0.029 | 0.185 | 0.068 | 2.711 | 0.007 | 0.046 | 0.321 | 30.21% |
|  | c’ | direct effect | 0.067 | 0.429 | 0.207 | 2.068 | 0.039 | 0.008 | 0.827 | 69.79% |
|  | c | total effect | 0.096 | 0.614 | 0.208 | 2.952 | 0.003 | 0.183 | 1.019 | 100.00% |
| 8 | ab | indirect effect | 0.020 | 0.125 | 0.060 | 2.078 | 0.038 | 0.009 | 0.25 | 20.62% |
|  | c’ | direct effect | 0.077 | 0.490 | 0.206 | 2.379 | 0.017 | 0.075 | 0.877 | 79.38% |
|  | c | total effect | 0.097 | 0.615 | 0.208 | 2.951 | 0.003 | 0.185 | 1.018 | 100.00% |
| 9 | ab | indirect effect | 0.013 | 0.081 | 0.047 | 1.727 | 0.084 | -0.005 | 0.18 | 13.54% |
|  | c’ | direct effect | 0.083 | 0.533 | 0.207 | 2.580 | 0.010 | 0.113 | 0.937 | 86.46% |
|  | c | total effect | 0.096 | 0.614 | 0.209 | 2.944 | 0.003 | 0.183 | 1.02 | 100.00% |

Abbreviations: POD, postoperative day

**Table S5. Path analysis of mediating effects of pain between surgical approach and walking difficulty on POD2 dichotomized by different cut-off points of pain scores.**

| M | Path | Effect | Result | | | Z | *P* | 95% CI | | Proportion of total effect |
| --- | --- | --- | --- | --- | --- | --- | --- | --- | --- | --- |
|  |  |  | *β* | Estimate | SE |  |  | lower | upper |  |
| 1 | ab | indirect effect | -0.004 | -0.02 | 0.030 | -0.681 | 0.496 | -0.087 | 0.029 | -2.88% |
|  | c’ | direct effect | 0.143 | 0.722 | 0.174 | 4.156 | <0.001 | 0.368 | 1.053 | 102.88% |
|  | c | total effect | 0.139 | 0.701 | 0.177 | 3.969 | <0.001 | 0.33 | 1.035 | 100.00% |
| 2 | ab | indirect effect | 0.017 | 0.084 | 0.044 | 1.918 | 0.055 | -0.012 | 0.169 | 12.23% |
|  | c’ | direct effect | 0.122 | 0.618 | 0.170 | 3.632 | <0.001 | 0.266 | 0.949 | 87.77% |
|  | c | total effect | 0.139 | 0.702 | 0.176 | 3.981 | <0.001 | 0.327 | 1.037 | 100.00% |
| 3 | ab | indirect effect | 0.030 | 0.152 | 0.053 | 2.874 | 0.004 | 0.040 | 0.251 | 21.43% |
|  | c’ | direct effect | 0.110 | 0.554 | 0.168 | 3.290 | 0.001 | 0.201 | 0.889 | 78.57% |
|  | c | total effect | 0.140 | 0.706 | 0.176 | 4.007 | <0.001 | 0.332 | 1.041 | 100.00% |
| **4** | **ab** | **indirect effect** | **0.044** | **0.221** | **0.069** | **3.179** | **0.001** | **0.088** | **0.357** | **32.12%** |
|  | c’ | direct effect | 0.093 | 0.471 | 0.163 | 2.897 | 0.004 | 0.134 | 0.777 | 67.88% |
|  | c | total effect | 0.137 | 0.692 | 0.176 | 3.927 | <0.001 | 0.325 | 1.034 | 100.00% |
| 5 | a*b | indirect effect | 0.037 | 0.186 | 0.070 | 2.662 | 0.008 | 0.049 | 0.325 | 27.01% |
|  | c’ | direct effect | 0.100 | 0.505 | 0.160 | 3.157 | 0.002 | 0.185 | 0.813 | 72.99% |
|  | c | total effect | 0.137 | 0.691 | 0.176 | 3.926 | <0.001 | 0.331 | 1.025 | 100.00% |
| 6 | ab | indirect effect | 0.033 | 0.168 | 0.071 | 2.361 | 0.018 | 0.035 | 0.312 | 23.91% |
|  | c’ | direct effect | 0.105 | 0.530 | 0.158 | 3.347 | 0.001 | 0.212 | 0.825 | 76.09% |
|  | c | total effect | 0.138 | 0.699 | 0.176 | 3.965 | <0.001 | 0.334 | 1.035 | 100.00% |
| 7 | ab | indirect effect | 0.027 | 0.134 | 0.070 | 1.901 | 0.057 | -0.006 | 0.279 | 19.42% |
|  | c’ | direct effect | 0.112 | 0.564 | 0.162 | 3.491 | <0.001 | 0.229 | 0.885 | 80.58% |
|  | c | total effect | 0.139 | 0.698 | 0.176 | 3.972 | <0.001 | 0.335 | 1.033 | 100.00% |
| 8 | ab | indirect effect | 0.018 | 0.091 | 0.056 | 1.622 | 0.105 | -0.02 | 0.205 | 12.77% |
|  | c’ | direct effect | 0.123 | 0.621 | 0.167 | 3.710 | <0.001 | 0.287 | 0.935 | 87.23% |
|  | c | total effect | 0.141 | 0.712 | 0.177 | 4.032 | <0.001 | 0.343 | 1.050 | 100.00% |
| 9 | ab | indirect effect | 0.009 | 0.047 | 0.038 | 1.233 | 0.217 | -0.027 | 0.126 | 6.43% |
|  | c’ | direct effect | 0.131 | 0.659 | 0.173 | 3.814 | <0.001 | 0.309 | 0.993 | 93.57% |
|  | c | total effect | 0.140 | 0.706 | 0.176 | 4.014 | <0.001 | 0.330 | 1.042 | 100.00% |

Abbreviations: POD, postoperative day

**Table S6. Path analysis of mediating effects of pain between surgical approach and** **walking difficulty on POD3 dichotomized by different cut-off points of pain scores.**

| M | Path | Effect | Result | | | Z | P | 95% CI | | Proportion of total effect |
| --- | --- | --- | --- | --- | --- | --- | --- | --- | --- | --- |
|  |  |  | *β* | Estimate | SE |  |  | lower | upper |  |
| 1 | ab | indirect effect | 0.003 | 0.016 | 0.031 | 0.500 | 0.617 | -0.054 | 0.072 | 2.05% |
|  | c’ | direct effect | 0.143 | 0.659 | 0.167 | 3.949 | <0.001 | 0.321 | 0.986 | 97.95% |
|  | c | total effect | 0.146 | 0.674 | 0.170 | 3.962 | <0.001 | 0.325 | 0.995 | 100.00% |
| 2 | ab | indirect effect | 0.028 | 0.127 | 0.058 | 2.201 | 0.028 | 0.002 | 0.239 | 19.18% |
|  | c’ | direct effect | 0.118 | 0.542 | 0.160 | 3.386 | 0.001 | 0.239 | 0.860 | 80.82% |
|  | c | total effect | 0.146 | 0.669 | 0.170 | 3.940 | <0.001 | 0.323 | 0.991 | 100.00% |
| **3** | **ab** | **indirect effect** | **0.045** | **0.208** | **0.074** | **2.825** | **0.005** | **0.056** | **0.353** | **30.82%** |
|  | c’ | direct effect | 0.101 | 0.464 | 0.155 | 3 | 0.003 | 0.172 | 0.760 | 69.18% |
|  | c | total effect | 0.146 | 0.672 | 0.170 | 3.956 | <0.001 | 0.326 | 0.997 | 100.00% |
| 4 | ab | indirect effect | 0.042 | 0.195 | 0.086 | 2.275 | 0.023 | 0.021 | 0.366 | 28.00% |
|  | c’ | direct effect | 0.108 | 0.496 | 0.150 | 3.314 | 0.001 | 0.208 | 0.799 | 72.00% |
|  | c | total effect | 0.150 | 0.691 | 0.170 | 4.053 | <0.001 | 0.343 | 1.009 | 100.00% |
| 5 | ab | indirect effect | 0.041 | 0.188 | 0.082 | 2.284 | 0.022 | 0.028 | 0.350 | 28.08% |
|  | c’ | direct effect | 0.105 | 0.483 | 0.147 | 3.28 | 0.001 | 0.187 | 0.782 | 71.92% |
|  | c | total effect | 0.146 | 0.671 | 0.170 | 3.935 | <0.001 | 0.323 | 0.990 | 100.00% |
| 6 | ab | indirect effect | 0.024 | 0.110 | 0.070 | 1.570 | 0.116 | -0.026 | 0.245 | 16.90% |
|  | c’ | direct effect | 0.118 | 0.541 | 0.155 | 3.493 | <0.001 | 0.227 | 0.844 | 83.10% |
|  | c | total effect | 0.142 | 0.651 | 0.170 | 3.829 | <0.001 | 0.307 | 0.971 | 100.00% |
| 7 | ab | indirect effect | 0 | -0.002 | 0.058 | -0.036 | 0.972 | -0.113 | 0.121 | 0.00% |
|  | c’ | direct effect | 0.142 | 0.651 | 0.159 | 4.092 | <0.001 | 0.326 | 0.959 | 100.00% |
|  | c | total effect | 0.142 | 0.649 | 0.170 | 3.819 | <0.001 | 0.301 | 0.973 | 100.00% |
| 8 | ab | indirect effect | 0 | -0.002 | 0.049 | -0.031 | 0.975 | -0.097 | 0.101 | 0.00% |
|  | c’ | direct effect | 0.147 | 0.675 | 0.164 | 4.128 | <0.001 | 0.360 | 0.993 | 100.00% |
|  | c | total effect | 0.147 | 0.674 | 0.171 | 3.947 | <0.001 | 0.330 | 0.999 | 100.00% |
| 9 | ab | indirect effect | -0.009 | -0.043 | 0.029 | -1.474 | 0.141 | -0.106 | 0.010 | -6.21% |
|  | c’ | direct effect | 0.154 | 0.709 | 0.169 | 4.189 | <0.001 | 0.367 | 1.039 | 106.21% |
|  | c | total effect | 0.145 | 0.665 | 0.171 | 3.901 | <0.001 | 0.319 | 0.997 | 100.00% |

Abbreviations: POD, postoperative day

# Table S7. Fit index based on two mediation effect models of activity limitation and walking difficulty on POD1 to POD3.

| Fit index |  | χ^2^ | *P* value | GFI | CFI | TLI | SRMR | RMSEA |
| --- | --- | --- | --- | --- | --- | --- | --- | --- |
| POD1 | Y1 | 0.501 | 0.479 | 1.000 | 1.000 | 1.002 | 0.004 | 0.000 |
|  | Y2 | 0.219 | 0.640 | 1.000 | 1.000 | 1.003 | 0.003 | 0.000 |
| POD2 | Y1 | 0.173 | 0.678 | 1.000 | 1.000 | 1.003 | 0.002 | 0.000 |
|  | Y2 | 1.362 | 0.243 | 1.000 | 1.000 | 0.999 | 0.007 | 0.018 |
| POD3 | Y1 | 0.044 | 0.834 | 1.000 | 1.000 | 1.003 | 0.001 | 0.000 |
|  | Y2 | 0.022 | 0.882 | 1.000 | 1.000 | 1.003 | 0.001 | 0.000 |

Y1, activity limitation; Y2, walking difficulty; Standard, thresholds of good model fit.

Abbreviations: GFI: Goodness-of-Fit Index; CFI: Comparative Fit Index; TLI: Tucker-Lewis Index; SRMR: Standardized Root Mean Square Residual; RMSEA: Root Mean Square Error of Approximation

# Table S8. Path analysis of mediating effects of pain between surgical approach and activity limitation/walking difficulty without missing data imputation.

| Variable | Path | Effect | Result | | | Z | P | 95% CI | | Proportion of total effect |
| --- | --- | --- | --- | --- | --- | --- | --- | --- | --- | --- |
|  |  |  | *β* | Estimate | SE |  |  | lower | upper |  |
| Y1 | ab | indirect effect | 0.039 | 0.206 | 0.048 | 4.265 | <0.001 | 0.108 | 0.306 | 54.17% |
|  | c’ | direct effect | 0.033 | 0.173 | 0.075 | 2.292 | 0.022 | 0.035 | 0.324 | 45.83% |
|  | c | total effect | 0.072 | 0.379 | 0.092 | 4.121 | <0.001 | 0.200 | 0.554 | 100.00% |
| Y2 | ab | indirect effect | 0.037 | 0.195 | 0.048 | 4.022 | <0.001 | 0.107 | 0.297 | 56.92% |
|  | c’ | direct effect | 0.028 | 0.151 | 0.076 | 1.983 | 0.047 | -0.001 | 0.290 | 43.08% |
|  | c | total effect | 0.065 | 0.346 | 0.090 | 3.831 | <0.001 | 0.16 | 0.517 | 100.00% |

Y1, activity limitation; Y2, walking difficulty

Abbreviations: FIML, full information maximum likelihood

# Table S9. Path analysis of mediating effects of pain between surgical approach and activity limitation/walking difficulty without propensity score.

| Variable | Path | Effect | Result | | | Z | P | 95% CI | | Proportion of total effect |
| --- | --- | --- | --- | --- | --- | --- | --- | --- | --- | --- |
|  |  |  | *β* | Estimate | SE |  |  | lower | upper |  |
| Y1 | ab | indirect effect | 0.034 | 0.177 | 0.044 | 3.989 | <0.001 | 0.09 | 0.267 | 53.13% |
|  | c’ | direct effect | 0.031 | 0.162 | 0.074 | 2.171 | 0.030 | 0.021 | 0.307 | 48.44% |
|  | c | total effect | 0.064 | 0.338 | 0.089 | 3.781 | <0.001 | 0.164 | 0.502 | 100.00% |
| Y2 | ab | indirect effect | 0.032 | 0.171 | 0.042 | 4.043 | <0.001 | 0.087 | 0.255 | 50.00% |
|  | c’ | direct effect | 0.032 | 0.172 | 0.072 | 2.382 | 0.017 | 0.035 | 0.313 | 50.00% |
|  | c | total effect | 0.064 | 0.342 | 0.087 | 3.936 | <0.001 | 0.177 | 0.509 | 100.00% |

Y1, activity limitation; Y2, walking difficulty

# Table S10. Path analysis of mediating effects of pain between surgical approach and activity limitation on POD1 without consideration of propensity scores using different cut-off points of pain scores.

| M | Path | Effect | Result | | | Z | P | 95% CI | | Proportion of total effect |
| --- | --- | --- | --- | --- | --- | --- | --- | --- | --- | --- |
|  |  |  | *β* | Estimate | SE |  |  | lower | upper |  |
| 1 | ab | indirect effect | -0.002 | -0.012 | 0.045 | -0.268 | 0.789 | -0.111 | 0.069 | -2.13% |
|  | c’ | direct effect | 0.096 | 0.595 | 0.199 | 2.983 | 0.003 | 0.207 | 0.974 | 102.13% |
|  | c | total effect | 0.094 | 0.583 | 0.207 | 2.817 | 0.005 | 0.175 | 0.985 | 100.00% |
| 2 | ab | indirect effect | 0.011 | 0.069 | 0.050 | 1.383 | 0.167 | -0.042 | 0.157 | 11.83% |
|  | c’ | direct effect | 0.082 | 0.511 | 0.198 | 2.572 | 0.010 | 0.117 | 0.885 | 88.17% |
|  | c | total effect | 0.093 | 0.579 | 0.207 | 2.803 | 0.005 | 0.172 | 0.983 | 100.00% |
| 3 | ab | indirect effect | 0.018 | 0.113 | 0.061 | 1.867 | 0.062 | -0.008 | 0.23 | 20.22% |
|  | c’ | direct effect | 0.071 | 0.442 | 0.194 | 2.273 | 0.023 | 0.068 | 0.831 | 79.78% |
|  | c | total effect | 0.089 | 0.555 | 0.207 | 2.686 | 0.007 | 0.155 | 0.963 | 100.00% |
| 4 | ab | indirect effect | 0.021 | 0.133 | 0.068 | 1.958 | 0.050 | -0.003 | 0.268 | 23.60% |
|  | c’ | direct effect | 0.068 | 0.419 | 0.192 | 2.181 | 0.029 | 0.071 | 0.805 | 76.40% |
|  | c | total effect | 0.089 | 0.553 | 0.207 | 2.672 | 0.008 | 0.151 | 0.966 | 100.00% |
| 5 | ab | indirect effect | 0.024 | 0.151 | 0.075 | 2.008 | 0.045 | 0.007 | 0.312 | 26.97% |
|  | c’ | direct effect | 0.065 | 0.401 | 0.193 | 2.075 | 0.038 | 0.03 | 0.771 | 73.03% |
|  | c | total effect | 0.089 | 0.551 | 0.207 | 2.669 | 0.008 | 0.147 | 0.96 | 100.00% |
| 6 | ab | indirect effect | 0.018 | 0.113 | 0.076 | 1.473 | 0.141 | -0.045 | 0.263 | 20.69% |
|  | c’ | direct effect | 0.069 | 0.428 | 0.194 | 2.211 | 0.027 | 0.04 | 0.809 | 79.31% |
|  | c | total effect | 0.087 | 0.541 | 0.207 | 2.609 | 0.009 | 0.13 | 0.94 | 100.00% |
| 7 | ab | indirect effect | 0.026 | 0.159 | 0.071 | 2.251 | 0.024 | 0.025 | 0.297 | 28.57% |
|  | c’ | direct effect | 0.065 | 0.402 | 0.195 | 2.061 | 0.039 | 0.003 | 0.78 | 71.43% |
|  | c | total effect | 0.091 | 0.561 | 0.206 | 2.722 | 0.006 | 0.156 | 0.966 | 100.00% |
| 8 | ab | indirect effect | 0.018 | 0.113 | 0.06 | 1.899 | 0.058 | -0.005 | 0.23 | 20.00% |
|  | c’ | direct effect | 0.072 | 0.449 | 0.199 | 2.259 | 0.024 | 0.063 | 0.835 | 80.00% |
|  | c | total effect | 0.09 | 0.563 | 0.207 | 2.721 | 0.007 | 0.154 | 0.969 | 100.00% |
| 9 | ab | indirect effect | 0.012 | 0.072 | 0.049 | 1.479 | 0.139 | -0.02 | 0.176 | 13.04% |
|  | c’ | direct effect | 0.08 | 0.496 | 0.202 | 2.451 | 0.014 | 0.098 | 0.904 | 86.96% |
|  | c | total effect | 0.092 | 0.567 | 0.206 | 2.748 | 0.006 | 0.154 | 0.975 | 100.00% |

Abbreviations: POD, postoperative day

# Table S11. Path analysis of mediating effects of pain between surgical approach and activity limitation on POD2 without consideration of propensity scores dichotomized by different cut-off points of pain scores.

| M | Path | Effect | Result | | | Z | P | 95% CI | | Proportion of total effect |
| --- | --- | --- | --- | --- | --- | --- | --- | --- | --- | --- |
|  |  |  | *β* | Estimate | SE |  |  | lower | upper |  |
| 1 | ab | indirect effect | -0.005 | -0.025 | 0.034 | -0.754 | 0.451 | -0.099 | 0.031 | -3.85% |
|  | c’ | direct effect | 0.135 | 0.685 | 0.167 | 4.107 | <0.001 | 0.340 | 1.017 | 103.85% |
|  | c | total effect | 0.130 | 0.660 | 0.169 | 3.913 | <0.001 | 0.316 | 0.99 | 100.00% |
| 2 | ab | indirect effect | 0.017 | 0.086 | 0.049 | 1.746 | 0.081 | -0.01 | 0.187 | 13.18% |
|  | c’ | direct effect | 0.112 | 0.571 | 0.161 | 3.554 | <0.001 | 0.245 | 0.883 | 86.82% |
|  | c | total effect | 0.129 | 0.657 | 0.168 | 3.902 | <0.001 | 0.316 | 0.985 | 100.00% |
| 3 | ab | indirect effect | 0.030 | 0.154 | 0.057 | 2.710 | 0.007 | 0.043 | 0.270 | 23.26% |
|  | c’ | direct effect | 0.099 | 0.503 | 0.159 | 3.169 | 0.002 | 0.186 | 0.818 | 76.74% |
|  | c | total effect | 0.129 | 0.657 | 0.167 | 3.933 | <0.001 | 0.320 | 0.978 | 100.00% |
| 4 | ab | indirect effect | 0.041 | 0.208 | 0.070 | 2.986 | 0.003 | 0.068 | 0.342 | 32.03% |
|  | c’ | direct effect | 0.087 | 0.444 | 0.151 | 2.948 | 0.003 | 0.146 | 0.740 | 67.97% |
|  | c | total effect | 0.128 | 0.652 | 0.167 | 3.895 | <0.001 | 0.314 | 0.974 | 100.00% |
| 5 | ab | indirect effect | 0.035 | 0.180 | 0.074 | 2.423 | 0.015 | 0.041 | 0.335 | 27.34% |
|  | c’ | direct effect | 0.093 | 0.473 | 0.151 | 3.132 | 0.002 | 0.190 | 0.773 | 72.66% |
|  | c | total effect | 0.128 | 0.653 | 0.168 | 3.897 | <0.001 | 0.313 | 0.978 | 100.00% |
| 6 | ab | indirect effect | 0.036 | 0.181 | 0.077 | 2.359 | 0.018 | 0.030 | 0.342 | 27.91% |
|  | c’ | direct effect | 0.093 | 0.474 | 0.149 | 3.192 | 0.001 | 0.191 | 0.769 | 72.09% |
|  | c | total effect | 0.129 | 0.655 | 0.168 | 3.900 | <0.001 | 0.313 | 0.976 | 100.00% |
| 7 | ab | indirect effect | 0.027 | 0.136 | 0.072 | 1.872 | 0.061 | -0.008 | 0.270 | 21.43% |
|  | c’ | direct effect | 0.099 | 0.501 | 0.149 | 3.362 | 0.001 | 0.216 | 0.804 | 78.57% |
|  | c | total effect | 0.126 | 0.637 | 0.168 | 3.790 | <0.001 | 0.298 | 0.963 | 100.00% |
| 8 | ab | indirect effect | 0.015 | 0.077 | 0.059 | 1.298 | 0.194 | -0.038 | 0.190 | 11.63% |
|  | c’ | direct effect | 0.114 | 0.576 | 0.157 | 3.670 | <0.001 | 0.264 | 0.876 | 88.37% |
|  | c | total effect | 0.129 | 0.653 | 0.168 | 3.888 | <0.001 | 0.306 | 0.980 | 100.00% |
| 9 | ab | indirect effect | 0.009 | 0.045 | 0.051 | 0.879 | 0.379 | -0.054 | 0.142 | 7.03% |
|  | c’ | direct effect | 0.119 | 0.604 | 0.162 | 3.729 | <0.001 | 0.277 | 0.929 | 92.97% |
|  | c | total effect | 0.128 | 0.649 | 0.168 | 3.863 | <0.001 | 0.303 | 0.974 | 100.00% |

Abbreviations: POD, postoperative day

# Table S12. Path analysis of mediating effects of pain between surgical approach and activity limitation on POD3 without consideration of propensity scores dichotomized by different cut-off points of pain scores.

| M | Path | Effect | Result | | | Z | P | 95% CI | | Proportion of total effect |
| --- | --- | --- | --- | --- | --- | --- | --- | --- | --- | --- |
|  |  |  | *β* | Estimate | SE |  |  | lower | upper |  |
| 1 | ab | indirect effect | 0.004 | 0.020 | 0.029 | 0.694 | 0.488 | -0.04 | 0.078 | 2.55% |
|  | c’ | direct effect | 0.153 | 0.703 | 0.157 | 4.473 | <0.001 | 0.397 | 1.012 | 97.45% |
|  | c | total effect | 0.157 | 0.723 | 0.16 | 4.514 | <0.001 | 0.417 | 1.041 | 100.00% |
| 2 | ab | indirect effect | 0.025 | 0.115 | 0.053 | 2.182 | 0.029 | 0.016 | 0.220 | 16.13% |
|  | c’ | direct effect | 0.13 | 0.596 | 0.153 | 3.900 | <0.001 | 0.301 | 0.881 | 83.87% |
|  | c | total effect | 0.155 | 0.712 | 0.160 | 4.451 | <0.001 | 0.406 | 1.020 | 100.00% |
| 3 | ab | indirect effect | 0.045 | 0.209 | 0.072 | 2.893 | 0.004 | 0.074 | 0.345 | 29.03% |
|  | c’ | direct effect | 0.11 | 0.506 | 0.147 | 3.432 | 0.001 | 0.218 | 0.796 | 70.97% |
|  | c | total effect | 0.155 | 0.714 | 0.159 | 4.490 | <0.001 | 0.415 | 1.023 | 100.00% |
| 4 | ab | indirect effect | 0.047 | 0.214 | 0.082 | 2.620 | 0.009 | 0.054 | 0.380 | 29.75% |
|  | c’ | direct effect | 0.111 | 0.512 | 0.145 | 3.534 | <0.001 | 0.235 | 0.797 | 70.25% |
|  | c | total effect | 0.158 | 0.726 | 0.159 | 4.559 | <0.001 | 0.427 | 1.043 | 100.00% |
| 5 | ab | indirect effect | 0.04 | 0.183 | 0.078 | 2.342 | 0.019 | 0.022 | 0.331 | 25.48% |
|  | c’ | direct effect | 0.117 | 0.539 | 0.144 | 3.735 | <0.001 | 0.259 | 0.821 | 74.52% |
|  | c | total effect | 0.157 | 0.722 | 0.16 | 4.521 | <0.001 | 0.424 | 1.039 | 100.00% |
| 6 | ab | indirect effect | 0.024 | 0.112 | 0.068 | 1.659 | 0.097 | -0.025 | 0.246 | 15.79% |
|  | c’ | direct effect | 0.128 | 0.591 | 0.145 | 4.062 | <0.001 | 0.302 | 0.879 | 84.21% |
|  | c | total effect | 0.152 | 0.703 | 0.16 | 4.406 | <0.001 | 0.397 | 1.017 | 100.00% |
| 7 | ab | indirect effect | 0.003 | 0.015 | 0.056 | 0.270 | 0.787 | -0.095 | 0.126 | 2.00% |
|  | c’ | direct effect | 0.147 | 0.678 | 0.15 | 4.526 | <0.001 | 0.399 | 0.985 | 98.00% |
|  | c | total effect | 0.15 | 0.693 | 0.159 | 4.355 | <0.001 | 0.382 | 1.010 | 100.00% |
| 8 | ab | indirect effect | 0.003 | 0.016 | 0.045 | 0.346 | 0.730 | -0.066 | 0.108 | 1.92% |
|  | c’ | direct effect | 0.153 | 0.703 | 0.154 | 4.558 | <0.001 | 0.405 | 1.012 | 98.08% |
|  | c | total effect | 0.156 | 0.719 | 0.159 | 4.513 | <0.001 | 0.419 | 1.036 | 100.00% |
| 9 | ab | indirect effect | -0.008 | -0.035 | 0.024 | -1.483 | 0.138 | -0.083 | 0.013 | -5.19% |
|  | c’ | direct effect | 0.162 | 0.745 | 0.157 | 4.736 | <0.001 | 0.443 | 1.047 | 105.19% |
|  | c | total effect | 0.154 | 0.710 | 0.159 | 4.469 | <0.001 | 0.409 | 1.024 | 100.00% |

Abbreviations: POD, postoperative day

# Table S13. Path analysis of mediating effects of pain between surgical approach and walking difficulty on POD1 without consideration of propensity scores dichotomized by different cut-off points of pain scores.

| M | Path | Effect | Result | | | Z | P | 95% CI | | Proportion of total effect |
| --- | --- | --- | --- | --- | --- | --- | --- | --- | --- | --- |
|  |  |  | *β* | Estimate | SE |  |  | lower | upper |  |
| 1 | ab | indirect effect | -0.002 | -0.012 | 0.047 | -0.259 | 0.796 | -0.12 | 0.070 | -2.70% |
|  | c’ | direct effect | 0.076 | 0.481 | 0.2 | 2.401 | 0.016 | 0.098 | 0.876 | 102.70% |
|  | c | total effect | 0.074 | 0.469 | 0.207 | 2.266 | 0.023 | 0.078 | 0.880 | 100.00% |
| 2 | ab | indirect effect | 0.011 | 0.069 | 0.05 | 1.386 | 0.166 | -0.04 | 0.160 | 14.67% |
|  | c’ | direct effect | 0.064 | 0.403 | 0.2 | 2.012 | 0.044 | 0.021 | 0.786 | 85.33% |
|  | c | total effect | 0.075 | 0.473 | 0.207 | 2.281 | 0.023 | 0.083 | 0.889 | 100.00% |
| 3 | ab | indirect effect | 0.018 | 0.112 | 0.06 | 1.861 | 0.063 | -0.007 | 0.226 | 25.35% |
|  | c’ | direct effect | 0.053 | 0.338 | 0.195 | 1.729 | 0.084 | -0.044 | 0.711 | 74.65% |
|  | c | total effect | 0.071 | 0.45 | 0.206 | 2.181 | 0.029 | 0.068 | 0.851 | 100.00% |
| 4 | ab | indirect effect | 0.02 | 0.124 | 0.064 | 1.946 | 0.052 | -0.004 | 0.249 | 28.57% |
|  | c’ | direct effect | 0.05 | 0.318 | 0.196 | 1.622 | 0.105 | -0.049 | 0.700 | 71.43% |
|  | c | total effect | 0.07 | 0.442 | 0.207 | 2.138 | 0.032 | 0.062 | 0.859 | 100.00% |
| 5 | ab | indirect effect | 0.023 | 0.143 | 0.071 | 2.014 | 0.044 | 0.008 | 0.291 | 32.86% |
|  | c’ | direct effect | 0.047 | 0.298 | 0.194 | 1.534 | 0.125 | -0.074 | 0.680 | 67.14% |
|  | c | total effect | 0.07 | 0.441 | 0.206 | 2.136 | 0.033 | 0.052 | 0.857 | 100.00% |
| 6 | ab | indirect effect | 0.017 | 0.106 | 0.072 | 1.476 | 0.140 | -0.040 | 0.254 | 24.29% |
|  | c’ | direct effect | 0.053 | 0.338 | 0.196 | 1.727 | 0.084 | -0.033 | 0.720 | 75.71% |
|  | c | total effect | 0.07 | 0.444 | 0.207 | 2.148 | 0.032 | 0.054 | 0.859 | 100.00% |
| 7 | ab | indirect effect | 0.024 | 0.154 | 0.069 | 2.244 | 0.025 | 0.023 | 0.285 | 33.33% |
|  | c’ | direct effect | 0.048 | 0.303 | 0.197 | 1.538 | 0.124 | -0.073 | 0.694 | 66.67% |
|  | c | total effect | 0.072 | 0.457 | 0.206 | 2.217 | 0.027 | 0.072 | 0.865 | 100.00% |
| 8 | ab | indirect effect | 0.018 | 0.115 | 0.06 | 1.91 | 0.056 | -0.003 | 0.231 | 25.00% |
|  | c’ | direct effect | 0.054 | 0.344 | 0.2 | 1.717 | 0.086 | -0.029 | 0.739 | 75.00% |
|  | c | total effect | 0.072 | 0.458 | 0.207 | 2.216 | 0.027 | 0.071 | 0.868 | 100.00% |
| 9 | ab | indirect effect | 0.011 | 0.069 | 0.045 | 1.554 | 0.120 | -0.021 | 0.161 | 15.07% |
|  | c’ | direct effect | 0.062 | 0.39 | 0.204 | 1.915 | 0.056 | 0.006 | 0.806 | 84.93% |
|  | c | total effect | 0.073 | 0.46 | 0.207 | 2.226 | 0.026 | 0.067 | 0.872 | 100.00% |

Abbreviations: POD, postoperative day

# Table S14. Path analysis of mediating effects of pain between surgical approach and walking difficulty on POD2 without consideration of propensity scores dichotomized by different cut-off points of pain scores.

| M | Path | Effect | Result | | | Z | *P* | 95% CI | | Proportion of total effect |
| --- | --- | --- | --- | --- | --- | --- | --- | --- | --- | --- |
|  |  |  | *β* | Estimate | SE |  |  | lower | upper |  |
| 1 | ab | indirect effect | -0.004 | -0.022 | 0.030 | -0.739 | 0.460 | -0.093 | 0.025 | -2.99% |
|  | c’ | direct effect | 0.138 | 0.689 | 0.164 | 4.211 | <0.001 | 0.366 | 0.995 | 102.99% |
|  | c | total effect | 0.134 | 0.667 | 0.165 | 4.031 | <0.001 | 0.348 | 0.976 | 100.00% |
| 2 | ab | indirect effect | 0.015 | 0.077 | 0.044 | 1.744 | 0.081 | -0.009 | 0.167 | 11.28% |
|  | c’ | direct effect | 0.118 | 0.591 | 0.159 | 3.705 | <0.001 | 0.287 | 0.896 | 88.72% |
|  | c | total effect | 0.133 | 0.668 | 0.165 | 4.056 | <0.001 | 0.352 | 0.976 | 100.00% |
| 3 | ab | indirect effect | 0.029 | 0.143 | 0.053 | 2.691 | 0.007 | 0.041 | 0.252 | 21.48% |
|  | c’ | direct effect | 0.106 | 0.528 | 0.159 | 3.325 | 0.001 | 0.224 | 0.820 | 78.52% |
|  | c | total effect | 0.135 | 0.671 | 0.165 | 4.072 | <0.001 | 0.353 | 0.981 | 100.00% |
| 4 | ab | indirect effect | 0.040 | 0.203 | 0.069 | 2.956 | 0.003 | 0.066 | 0.335 | 30.53% |
|  | c’ | direct effect | 0.091 | 0.455 | 0.152 | 2.998 | 0.003 | 0.162 | 0.739 | 69.47% |
|  | c | total effect | 0.131 | 0.658 | 0.165 | 3.976 | <0.001 | 0.341 | 0.969 | 100.00% |
| 5 | ab | indirect effect | 0.034 | 0.171 | 0.071 | 2.413 | 0.016 | 0.038 | 0.317 | 25.95% |
|  | c’ | direct effect | 0.097 | 0.486 | 0.152 | 3.203 | 0.001 | 0.196 | 0.763 | 74.05% |
|  | c | total effect | 0.131 | 0.656 | 0.165 | 3.969 | <0.001 | 0.343 | 0.965 | 100.00% |
| 6 | ab | indirect effect | 0.033 | 0.164 | 0.069 | 2.363 | 0.018 | 0.028 | 0.314 | 24.81% |
|  | c’ | direct effect | 0.100 | 0.500 | 0.15 | 3.325 | 0.001 | 0.209 | 0.780 | 75.19% |
|  | c | total effect | 0.133 | 0.664 | 0.165 | 4.014 | <0.001 | 0.349 | 0.974 | 100.00% |
| 7 | ab | indirect effect | 0.024 | 0.122 | 0.065 | 1.866 | 0.062 | -0.008 | 0.242 | 18.18% |
|  | c’ | direct effect | 0.108 | 0.542 | 0.151 | 3.593 | <0.001 | 0.247 | 0.827 | 81.82% |
|  | c | total effect | 0.132 | 0.663 | 0.165 | 4.011 | <0.001 | 0.346 | 0.973 | 100.00% |
| 8 | ab | indirect effect | 0.014 | 0.070 | 0.054 | 1.288 | 0.198 | -0.036 | 0.179 | 10.37% |
|  | c’ | direct effect | 0.121 | 0.607 | 0.157 | 3.854 | <0.001 | 0.304 | 0.894 | 89.63% |
|  | c | total effect | 0.135 | 0.677 | 0.166 | 4.070 | <0.001 | 0.355 | 0.990 | 100.00% |
| 9 | ab | indirect effect | 0.007 | 0.033 | 0.038 | 0.861 | 0.389 | -0.043 | 0.110 | 5.22% |
|  | c’ | direct effect | 0.127 | 0.637 | 0.162 | 3.928 | <0.001 | 0.324 | 0.941 | 94.78% |
|  | c | total effect | 0.134 | 0.670 | 0.165 | 4.053 | <0.001 | 0.352 | 0.981 | 100.00% |

Abbreviations: POD, postoperative day

# Table S15. Path analysis of mediating effects of pain between surgical approach and walking difficulty on POD3 without consideration of propensity scores dichotomized by different cut-off points of pain scores.

| M | Path | Effect | Result | | | Z | *P* | 95% CI | | Proportion of total effect |
| --- | --- | --- | --- | --- | --- | --- | --- | --- | --- | --- |
|  |  |  | *β* | Estimate | SE |  |  | lower | upper |  |
| 1 | ab | indirect effect | 0.004 | 0.020 | 0.028 | 0.719 | 0.472 | -0.039 | 0.075 | 2.53% |
|  | c’ | direct effect | 0.154 | 0.702 | 0.155 | 4.522 | <0.001 | 0.412 | 0.992 | 97.47% |
|  | c | total effect | 0.158 | 0.722 | 0.158 | 4.570 | <0.001 | 0.422 | 1.020 | 100.00% |
| 2 | ab | indirect effect | 0.026 | 0.117 | 0.053 | 2.214 | 0.027 | 0.019 | 0.226 | 16.46% |
|  | c’ | direct effect | 0.132 | 0.601 | 0.151 | 3.988 | <0.001 | 0.330 | 0.889 | 83.54% |
|  | c | total effect | 0.158 | 0.718 | 0.158 | 4.543 | <0.001 | 0.419 | 1.013 | 100.00% |
| 3 | ab | indirect effect | 0.045 | 0.205 | 0.070 | 2.926 | 0.003 | 0.075 | 0.341 | 28.48% |
|  | c’ | direct effect | 0.113 | 0.515 | 0.146 | 3.533 | <0.001 | 0.231 | 0.789 | 71.52% |
|  | c | total effect | 0.158 | 0.721 | 0.158 | 4.574 | <0.001 | 0.422 | 1.019 | 100.00% |
| 4 | ab | indirect effect | 0.046 | 0.208 | 0.078 | 2.667 | 0.008 | 0.054 | 0.375 | 28.40% |
|  | c’ | direct effect | 0.116 | 0.527 | 0.144 | 3.653 | <0.001 | 0.252 | 0.804 | 71.60% |
|  | c | total effect | 0.162 | 0.736 | 0.158 | 4.667 | <0.001 | 0.440 | 1.035 | 100.00% |
| 5 | ab | indirect effect | 0.04 | 0.183 | 0.077 | 2.387 | 0.017 | 0.024 | 0.328 | 25.48% |
|  | c’ | direct effect | 0.117 | 0.533 | 0.142 | 3.750 | <0.001 | 0.266 | 0.805 | 74.52% |
|  | c | total effect | 0.157 | 0.716 | 0.158 | 4.529 | <0.001 | 0.422 | 1.017 | 100.00% |
| 6 | ab | indirect effect | 0.024 | 0.111 | 0.065 | 1.700 | 0.089 | -0.021 | 0.242 | 15.79% |
|  | c’ | direct effect | 0.128 | 0.585 | 0.143 | 4.093 | <0.001 | 0.306 | 0.855 | 84.21% |
|  | c | total effect | 0.152 | 0.696 | 0.157 | 4.416 | <0.001 | 0.400 | 0.994 | 100.00% |
| 7 | ab | indirect effect | 0.004 | 0.017 | 0.055 | 0.307 | 0.759 | -0.091 | 0.125 | 2.63% |
|  | c’ | direct effect | 0.148 | 0.676 | 0.148 | 4.576 | <0.001 | 0.393 | 0.963 | 97.37% |
|  | c | total effect | 0.152 | 0.693 | 0.157 | 4.402 | <0.001 | 0.395 | 0.991 | 100.00% |
| 8 | ab | indirect effect | 0.004 | 0.017 | 0.045 | 0.375 | 0.708 | -0.073 | 0.105 | 2.53% |
|  | c’ | direct effect | 0.154 | 0.703 | 0.152 | 4.625 | <0.001 | 0.416 | 0.997 | 97.47% |
|  | c | total effect | 0.158 | 0.720 | 0.157 | 4.574 | <0.001 | 0.418 | 1.017 | 100.00% |
| 9 | ab | indirect effect | -0.008 | -0.037 | 0.026 | -1.449 | 0.147 | -0.092 | 0.014 | -5.13% |
|  | c’ | direct effect | 0.164 | 0.749 | 0.155 | 4.831 | <0.001 | 0.450 | 1.033 | 105.13% |
|  | c | total effect | 0.156 | 0.712 | 0.158 | 4.517 | <0.001 | 0.413 | 1.011 | 100.00% |

Abbreviations: POD, postoperative day

**Table S16 Patient symptom scores from postoperative day 1 to day 3**

| **Postoperative days** | **Symptom** | **Mean** | **SD** | **Median** | **Q1** | **Q 3** |
| --- | --- | --- | --- | --- | --- | --- |
| 1 | pain | 5.05 | 2.51 | 5 | 3 | 7 |
|  | cough | 3.22 | 2.33 | 3 | 1 | 5 |
|  | shortness of breath | 3.34 | 2.70 | 3 | 1 | 5 |
|  | disturbed sleep | 3.84 | 2.81 | 4 | 2 | 6 |
|  | fatigue | 4.27 | 2.67 | 4 | 2 | 6 |
|  | drowsiness | 4.48 | 2.79 | 5 | 2 | 7 |
|  | distress | 3.46 | 2.77 | 3 | 1 | 5 |
| 2 | pain | 4.55 | 2.40 | 4 | 3 | 6 |
|  | cough | 3.46 | 2.17 | 3 | 2 | 5 |
|  | shortness of breath | 3.42 | 2.44 | 3 | 2 | 5 |
|  | disturbed sleep | 3.69 | 2.58 | 3 | 2 | 5 |
|  | fatigue | 3.51 | 2.38 | 3 | 2 | 5 |
|  | drowsiness | 3.19 | 2.39 | 3 | 1 | 5 |
|  | distress | 3.02 | 2.51 | 3 | 1 | 5 |
| 3 | pain | 3.71 | 2.22 | 3 | 2 | 5 |
|  | cough | 3.57 | 2.19 | 3 | 2 | 5 |
|  | shortness of breath | 3.29 | 2.24 | 3 | 2 | 5 |
|  | disturbed sleep | 3.33 | 2.38 | 3 | 2 | 5 |
|  | fatigue | 3.24 | 2.18 | 3 | 2 | 5 |
|  | drowsiness | 2.79 | 2.07 | 3 | 1 | 4 |
|  | distress | 2.68 | 2.27 | 2 | 1 | 4 |

Note: PRO data were derived from the Pulmonary Surgery Perioperative Symptom Assessment (PSA-Lung) scale, which assesses seven postoperative symptoms and two functional states. Q1: The 25th percentile; Q3: The 75th percentile.


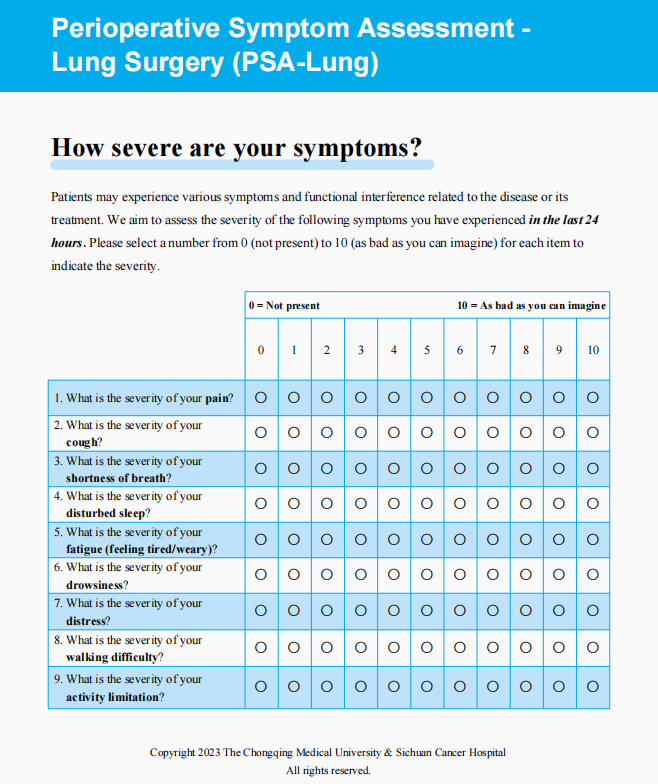


# Figure S1. Perioperative Symptom Assessment-Lung Surgery (PSA-Lung).

A B


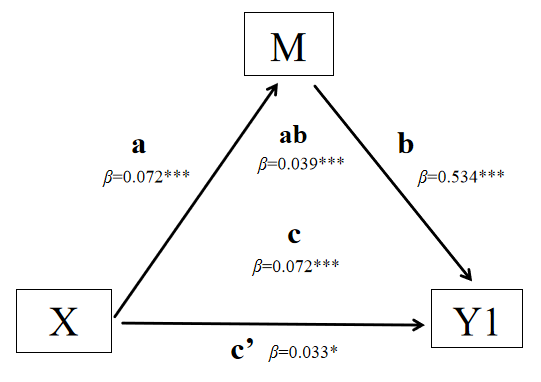

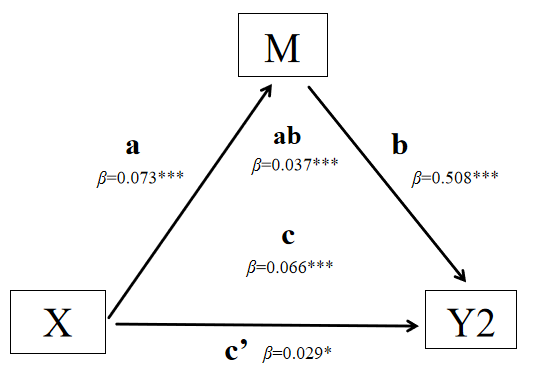


# Figure S2. Mediation effects identified by the LSEM.

X: Surgical approach (single-port VATS versus multi-port VATS/thoracotomy)

M: Pain (as a continuous variable)

Y: Functional status (activity limitation [Y1], walking difficulty [Y2])

Mediated effects are the product of a and b coefficients (ab). All values are standardized.

*: *p* < 0.05; **: *p* < 0.01; ***: *p* < 0.001

Abbreviations: LSEM，longitudinal structural equation model
